# Relationship between Fetuin A, Vascular Calcification and Fracture Risk in Dialysis Patients

**DOI:** 10.1371/journal.pone.0158789

**Published:** 2016-07-11

**Authors:** Hung Yuan Chen, Yen Ling Chiu, Shih Ping Hsu, Mei Fen Pai, Ju Yeh Yang, Yu Sen Peng

**Affiliations:** 1 Division of Nephrology, Department of Internal Medicine, Far Eastern Memorial Hospital, New Taipei City, Taiwan; 2 Division of Nephrology, Department of Internal Medicine, National Taiwan University Hospital, National Taiwan University College of Medicine, Taipei, Taiwan; Klinikum rechts der Isar—Technical University Munich—TUM, GERMANY

## Abstract

**Background:**

Fractures are a common morbidity that lead to worse outcomes in dialysis patients. Fetuin A inhibits vascular calcification (VC), potentially promotes bone mineralization and its level positively correlates with bone mineral density in the general population. On the other hand, the presence of VC is associated with low bone volume in dialysis patients. Whether the fetuin A level and VC can predict the occurrence of fractures in dialysis patients remains unknown.

**Methods:**

We performed this prospective, observational cohort study including 685 dialysis patients (629 hemodialysis and 56 peritoneal dialysis) from a single center in Taiwan for a median follow-up period of 3.4 years. The baseline fetuin A level and status of presence of aortic arch calcification (VC) and incidence of major fractures (hip, pelvis, humerus, proximal forearm, lower leg or vertebrae) were assessed using adjusted Cox proportional hazards models, recursive partitioning analysis and competing risk models.

**Results:**

Overall, 177 of the patients had major fractures. The incidence rate of major fractures was 3.29 per 100 person-years. In adjusted analyses, the patients with higher baseline fetuin A levels had a lower incidence of fractures (adjusted hazard ratio (HR), 0.3; 95% CI, 0.18‒0.5, fetuin A tertile 3 *vs*. tertile 1 and HR, 0.52; 95% CI, 0.34‒0.78, tertile 2 *vs*. tertile 1). The presence of aortic arch calcification (VC) independently predicted the occurrence of fractures (adjusted HR, 1.95; 95% CI, 1.34‒2.84) as well. When accounting for death as an event in competing risk models, the patients with higher baseline fetuin A levels remained to have a lower incidence of fractures (SHR, 0.31; 95% CI, 0.17‒0.56, fetuin A tertile 3 *vs*. tertile 1 and 0.51; 95% CI, 0.32‒0.81, tertile 2 *vs*. tertile 1).

**Interpretations:**

Lower baseline fetuin A levels and the presence of VC were independently linked to higher risk of incident fractures in prevalent dialysis patients.

## Introduction

Patients with chronic kidney disease (CKD), especially those undergoing dialysis, have unique mineral and endocrine disturbances which result in altered bone structure and function. It has been shown that patients undergoing dialysis have higher rates of bone fractures compared to the general population.[1, 2] In addition, patients experiencing a major bone fracture (e.g. hip fracture) have been reported to have a remarkable increase in subsequent disability, death and hospitalization.[3–5] Several major risk factors such as abnormal intact parathyroid hormone (iPTH) levels, heavy comorbidity burden, sarcopenia, increased susceptibility to falls, and polypharmacy can increase the likelihood of fractures in dialysis patients.[2, 6, 7] However, the link between fractures and vascular calcification (VC), another major component of mineral bone disorders in CKD patients, is as yet uncertain. In CKD and dialysis patients, VC has been shown to correlate with low trabecular bone volume and indices of low bone turnover.[8, 9] Adragao *et al* described an association between low bone volume and coronary calcifications in patients who were on dialysis for more than 6 years.[10] Of note, VC has a strong correlation with low bone volume in CKD patients, however little is known about the interrelationship between VC and fractures in dialysis patients.

Fetuin A is a glycoprotein synthesized in the liver and expressed in the extracellular space. It is a well-known inhibitor of VC in dialysis patients,[11] and it has been reported to promote bone mineralization in vitro.[12, 13] The role of fetuin A in tissue mineralization serving as a “mineral chaperone” has been proposed[14, 15]. In a landmark study, fetuin A colocalizes with matrix vesicles (MVs) which are secreted by human vascular smooth muscle cells (VSMCs) and is specifically loaded into MVs. These findings further strengthen the concerns between fetuin A and VC intracellularly[16]. However, its role in bone mineralization is understudied. In fetuin A knock-out mice, the trabecular bone mass and microstructure of cortical bone are unaffected by the absence of fetuin A; nevertheless, there is excess mineralization of the growth plate of long bone which causes short limbs[17]. In humans clinical study, the relationship between serum fetuin A level and bone mineral density (BMD) was investigated in 3075 well-functioning elderly persons, and the results showed that higher fetuin A levels were independently associated with higher BMD among women.[18] Nevertheless, a subsequent study showed no evidence of an association between fetuin A and the risk of clinical fractures.[19] These interesting but inconsistent findings prompted us to conduct this study in dialysis patients to investigate the connection between VC and bone volume and the potential link between the fetuin A and the risk of fractures as well. Therefore, the aim of this prospective, observational study was to test the hypothesis that dialysis patients with either lower fetuin A levels or VC would have a higher risk of incident fractures.

## Materials and Methods

### Subjects

**Fig 1:**
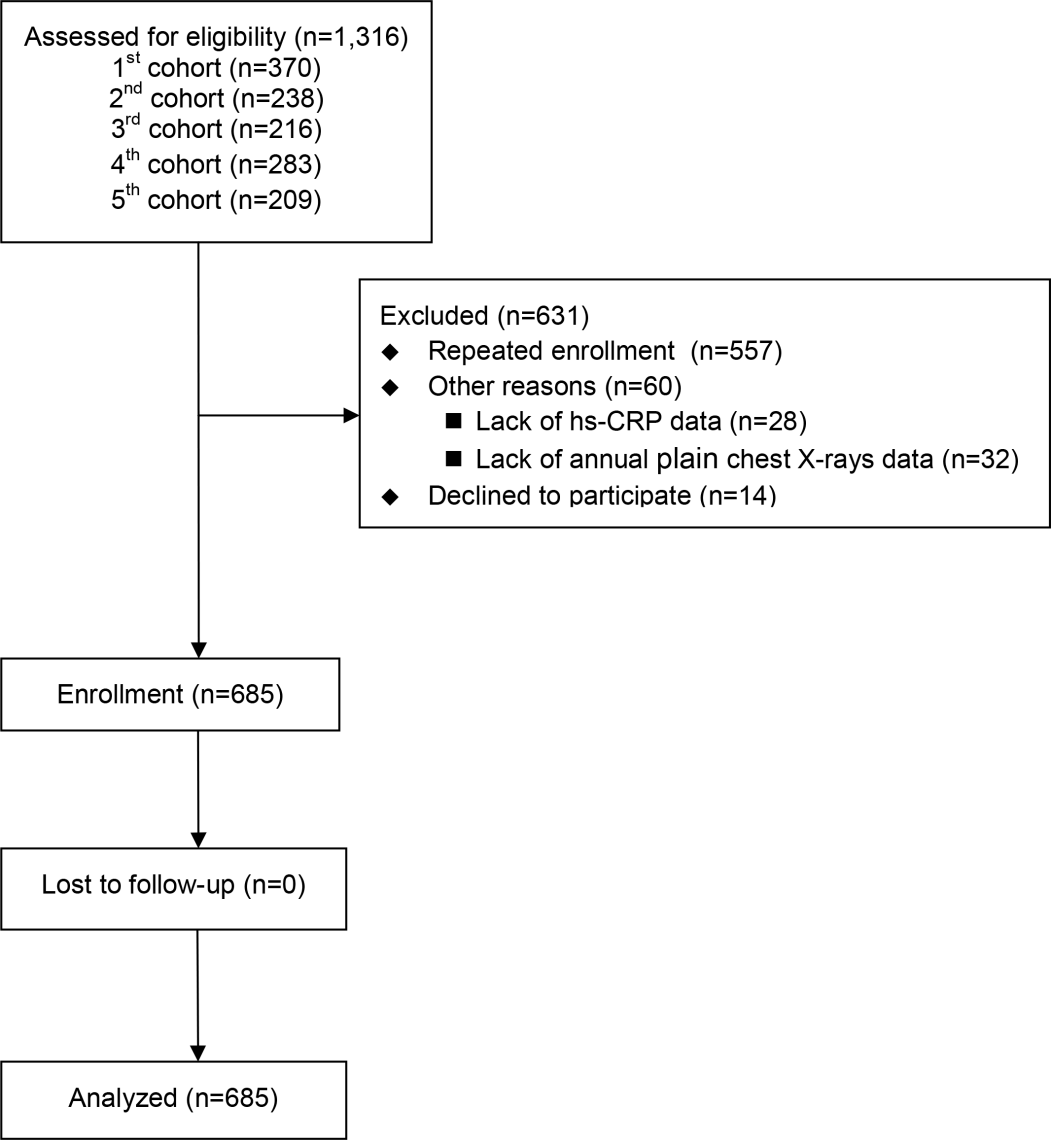
Study flow diagram.

This was a prospective study performed using five pooled patient cohorts. The first three cohorts were composed of 370, 238, and 216 prevalent hemodialysis (HD) patients, respectively, the fourth was composed of 220 HD patients and 63 peritoneal dialysis (PD) patients and the fifth was 209 HD patients. These patient cohorts have been described previously in more detail. [20–23] In brief, the five cohorts were collected prospectively to understand the associations between serum fetuin A level, inflammatory markers (such as, high sensitivity C-reactive protein (hs-CRP) and lipid profiles with specific outcomes in prevalent dialysis patients, at the Far Eastern Memorial Hospital from 2007 to 2014. All patients had baseline data on fetuin A at entry. The exclusion criteria of the five cohorts were as follows: (1) active infection; (2) recent hospitalization within 3 months; (3) psychotic illness or other communication problems; (4) active malignancy; (5) younger than 20 years; (6) receiving HD or PD for less than 3 months; and (7) patients’ refusal. In the flow diagram, we have clearly shown the reasons of exclusion in this cohort study (Fig 1). Before initiating this prospective study, we have re-evaluated all the participants in the five cohorts about the wills of being analyzed for the pre-specified outcome (fracture). For the most part, the exclusion of participants was due to repeated enrollment (88%), only 14 patients (1%) declined to participate this prospective analysis and none of the participants in the cohort were excluded because of missing fetuin A data. The final numbers of enrolled participants from the five cohorts are 354, 18, 117, 163 and 33 respectively.

All of the subjects provided written informed consent, the study complied with the World Medical Association Declaration of Helsinki—Ethical Principles for Medical Research Involving Human Subjects, and the Ethics Committee of Far Eastern Memorial Hospital approved the study protocol (ClinicalTrials.gov; NCT01457625).

In total, 685 patients (mean age, 59±13 years; 348 women) who underwent prevalent HD (629) and PD (56) at the Far Eastern Memorial Hospital, Taiwan, were enrolled from February 2007 (first cohort), March 2009 (second cohort), March 2011 (third cohort), March 2013 (fourth cohort) and September 2014 (fifth cohort). The median dialysis vintage before recruitment was 2.5 years (range, 0.4–26.5 years).

### Measurement of serum fetuin A concentrations

Serum fetuin A levels in the five cohorts were measured using three types of highly sensitive, two-site enzyme-linked immunoassays (GenWay Biotech, Inc., San Diego, CA, USA; Adipo Bioscience, Inc., Santa Clara, CA, USA and R&D Systems, Inc., Minneapolis, MN, USA). The intra-assay coefficients of variation were 4.1%, 4.0% and 4.0%, respectively, and the inter-assay coefficients of variation were 6.2%, 6% and 6.4%, respectively. The linear measurement ranges of the assays for human fetuin A levels were 0.002‒2.5 g/L and 0.003‒2.5 g/L and 0.002‒2.5 g/L, respectively. Blood samples for the measurement of fetuin A levels were obtained once on recruitment and were immediately centrifuged and stored at −70°C until the time of the assay.

### Measurements of clinical parameters

Demographic data, a concurrent medical history of CV disease and smoking status were recorded. Venous blood was sampled in the morning after an overnight fast of more than 8 hours before the patient’s mid-week dialysis session in the HD patients, or before the first daily dwell of dialysate in the PD patients. Intact PTH levels were determined by immunoassay (Roche Modular E170 analyzer). The hs-CRP levels were determined using the immunonephelometric method using a Tina-quant CRP (Latex) ultrasensitive assay (D & P Modular Analyzer, Roche Diagnostics GmbH, Mannheim, Germany). The geriatric nutritional risk index (GNRI) was calculated by the following formula: GNRI = [14.89 × albumin level(g/dL)] + [41.7 ×body weight/WLo], where WLo is the ideal body weight calculated from the Lorentz equation. The GNRI has previously been validated in dialysis patients, and a higher GNRI indicates better nutritional status.[24]

### Outcomes

The primary outcome was the incidence of major fractures, which was defined as a new symptomatic fracture of the hip, pelvis, humerus, distal forearm, lower leg or vertebrae that occurred during follow-up. The occurrence of a major fracture was assessed by a clinical diagnosis (either from inpatient chart review or outpatient medical records), and concurrent roentgenogram, ordered for a suspicious fracture, with defined evidence of a fracture in the formal roentgenogram report by a radiologist. The outcome information was centrally assessed by trained clinicians, nephrologists and radiologists.

Follow-up started from February 2007 (first cohort entry), March 2009 (second cohort entry), March 2011 (third cohort entry), March 2013 (fourth cohort entry), and September 2014 (fifth cohort entry) and censored on the date of a major fracture, at the end of the study (July 31, 2015), on the date of death or renal transplantation or at the time the patients were transferred to other dialysis facilities and were no longer followed up, whichever came first.

### Assessment of VC

We defined the presence of VC as the presence of aortic arch calcification on a posterior-anterior plain chest X-ray at the entry of study. All of the participants received routine annual posterior-anterior plain chest X-rays in our hospital. Two trained physicians blinded to the patients’ clinical data reviewed plain chest X-rays performed before study enrollment to assess the presence of aortic arch calcification.

### Statistical analysis

Continuous data were presented as mean ± SD or median (interquartile range (IQR)), and categorical data were reported as percentages. Differences in baseline characteristics and biochemical parameters between the HD and PD patients and subjects with/without VC were compared using the Student’s *t* test and Mann-Whitney U test. Similarly, differences in baseline characteristics and biochemical parameters among the patients within the fetuin A tertiles were compared using ANOVA and the Kruskal-Wallis H test, as appropriate. The chi-square test was used for categorical variables.

Since the fetuin A level was not normally distributed in the dialysis patients (P<0.001 by either Kolmogorov-Smirnov or Shapiro-Wilk Test), we constructed plots of the fetuin A levels and crude hazard ratios (HRs) of the incident major fractures using the *Lowess* function. The results revealed a non-linear relationship, suggesting the need for stratification of the patients into tertiles according to their fetuin A level for outcome analysis, which we then performed (S1 Fig). The primary predictor variables were the fetuin A level in each tertile: patients with a fetuin A level between 0.11‒0.35 g/L were in tertile 1, between 0.35‒0.65 g/L in tertile 2 and between 0.66‒1.89 g/L in tertile 3.

Owing to the non-linear relationship between the fetuin A levels and HRs of the incident major fractures, we performed the outcome analysis in two ways: (1) to construct an algorithm for stratifying the major fracture risks in dialysis patients with recursive partitioning analysis (RPA) [25]. We performed the RPA in order to repeatedly divide patients into subgroups whether they had major fracture or not. It ideally provided a nonparametric discriminating tree for discriminating the power of risk factors of major fracture. Once RPA selected the tree, we only selected each of the splits identified with statistical criterion of *P <* 0.01 for outcomes (incident major fracture). Any split that did not meet this criterion was deleted. The final nodes were then compared. Kaplan-Meier graphs were presented for the final set of prognostic groups; (2) and standard Cox proportional hazard models. We used the “Enter” method to analyze the HR of each primary predictor variable in the model. For each fracture outcome, we first adjusted for gender, age, dialysis vintage, previous fracture history, diabetes mellitus (DM) status/hypertension status, dialysis modality, smoking/alcohol status and patient cohort (Model 1). Additional models were further adjusted for factors potentially associated with the risk of fractures that may have been confounders of the association between fetuin A level and fractures. Model 2 was adjusted for the covariates in Model 1 as well as hemoglobin level, nutritional status (GNRI), iPTH, calcium phosphate product (CaXP) and hs-CRP level. In Model 3, we also adjusted for the presence of aortic arch calcification. Sensitivity analyses were performed to test the robustness of our findings. Analyses of fracture outcome were performed while accounting for the competing event of death using the method of Fine and Gray[26], because death may be an informative censoring event. Owing to the pathophysiological link between fetuin A and VC, we further explored the potential interaction between fetuin A and VC in the prediction of incident fractures. All of the statistical analyses were performed using SPSS software version 19.0 (SPSS, Inc., Chicago, IL, USA) and Stata IC, version 14 (StataCorp, College Station, TX). A P value less than 0.05 was considered to be statistically significant.

## Results

### The baseline characteristics of the participants

The baseline characteristics of all of the participants, those in the fetuin A tertiles are summarized in Table 1. The patients in the fetuin A tertiles had different age, body mass index (BMI) and nutritional status (Table 1). And the baseline characteristics of the participants receiving HD or PD and with/without VC are summarized in the Table 2. In general, more female patients received PD, fewer patients undergoing PD had diabetes, and those undergoing PD were younger, had lower hemoglobin and albumin levels, higher BMI, creatinine and fetuin A levels (Table 2). Patients with aortic arch calcification were older, had longer dialysis vintage, had higher levels of hs-CRP and lower fetuin A levels (Table 2).

**Table 1:** Baseline characteristics of the all patients and the patients by fetuin A tertile.

	All patients	Fetuin A tertile 1(0.11‒0.35)	Fetuin A tertile 2(0.35‒0.65)	Fetuin A tertile 3(0.66‒1.89)	P value
	n = 685	n = 228	n = 229	n = 228	
Age (years)	59 ± 13	61 ± 12	59 ±13	56 ±13	0.001
Female gender (%)	51	49	52	52	0.7
Diabetes mellitus (%)	47	51	47	44	0.3
Dialysis vintage (years)	2.5 (1.5, 4.4)	2.6 (1.9,4.6)	2.4 (1.6,4.6)	2.5 (1.3,4.2)	0.1
History of hypertension (%)	78	81	76	78	0.4
History of previous fracture (%)	16	21	14	14	0.2
Systolic BP (mmHg)	144 ± 31	145 ± 31	146±42	146 ±72	0.3
Diastolic BP (mmHg)	84 ± 12	84 ± 13	86 ±18	83 ± 14	0.2
BMI (kg/m^2^)	23.1 ± 3.9	22.3 ± 3.9	23.3 ± 3.8	23.8 ± 3.8	<0.001
Laboratory data					
Hemoglobin (g/dL)	11.0 ± 1.4	10.9 ± 1.5	11.1±1.4	10.9 ± 1.4	0.4
Cre (mg/dL)	10.9 ± 2.4	10.6 ± 2.2	10.9 ± 2.4	11.1 ± 2.7	0.1
K (mmol/L)	4.7 ± 0.8	4.8 ± 0.7	4.6 ±0.8	4.6± 0.8	0.1
Ca (mg/dL); corrected	9.2 ± 0.7	9.1 ± 0.7	9.3 ± 0.7	9.3 ± 0.7	0.01
P (mg/dL)	5.3 ± 1.4	5.2 ± 1.4	5.2 ± 1.3	5.4 ± 1.4	0.5
CaxP	49 ± 14	48 ± 13	48 ± 13	50 ± 14	0.2
iPTH (pg/mL)	257 (123, 485)	254 (130, 450)	298 (125, 580)	240 (114, 463)	0.5
hs-CRP (mg/L)	3.1 (1.1, 7.6)	3.4 (1.1, 8.4)	3.0 (1.1, 7.3)	2.9 (1.2, 7.2)	0.5
Albumin (g/L)	4.1 ± 0.4	4.0 ± 0.4	4.1±0.4	4.1 ± 0.4	0.3
GNRI	104.3 ± 9.6	102.3 ± 10	104.5 ± 8.9	106.1 ± 9.6	<0.001
Medications (%)					
ESA	91	91	91	90	0.3
Active Vitamin D3	45	46	43	45	0.3
Phosphorus binder	86	87	84	87	0.2
Calcium-containing	64	64	65	63	0.1
Non-calcium-containing	22	23	19	24	0.2
Medications for osteoporosis	0.5	0.4	0.8	0.4	0.3
Anti-hypertensive agents	55	55	60	52	0.2

Abbreviations: CVD, cardiovascular disease; BP, blood pressure; Cre, creatinine; BMI, body mass index; CaxP, calcium phosphate product; iPTH, intact parathyroid hormone; hs-CRP, high-sensitive C-reactive protein; GNRI, geriatric nutritional risk index; ESA, erythropoiesis-stimulating agents.

*Note*: Conversion factors for units: hemoglobin in g/dL to g/L, ×10; serum calcium in mg/dL to mmol/L, ×0.2495; serum phosphate in mg/dL to mmol/L, ×0.3229; serum albumin in g/dL to g/L, ×10. No conversion is necessary for serum iPTH in pg/mL and ng/L; serum potassium in mEq/L and mmol/L.

**Table 2:** Baseline characteristics of the patients undergoing hemodialysis (HD) and peritoneal dialysis (PD) and patients with and without vascular calcification (VC).

	HD patients	PD patients	P value	Patients with VC	Patients without VC	P value
	n = 629	n = 56		n = 365	n = 320	
Age (years)	59 ±12	53 ±13	0.001	64 ± 11	53 ± 12	<0.001
Female gender (%)	49	69	0.004	56	45	0.004
Diabetes mellitus (%)	50	27	0.001	56	38	<0.001
Dialysis vintage (years)	2.4 (1.4, 4.6)	2.6 (1.0, 5.4)	0.9	2.9 (1.6, 5.0)	2.2 (1.2, 4.2)	0.01
History of hypertension (%)	79	85	0.3	83	73	0.001
History of previous fracture (%)	21	31	0.1	20	14	0.08
Systolic BP (mmHg)	146±43	147 ±50	0.1	147 ±50	146 ±47	0.2
Diastolic BP (mmHg)	85 ±13	83 ± 19	0.2	84 ± 20	82 ±16	0.1
BMI (kg/m^2^)	23.0 ± 3.9	23.9 ± 3.1	0.02	23.3 ± 4.0	22.9 ± 3.7	0.2
Laboratory data						
Hemoglobin (g/dL)	11.0 ±1.4	10.5 ± 1.2	0.006	11.1 ± 1.4	10.9 ± 1.4	0.05
Cre (mg/dL)	10.7± 3.8	11.3 ± 3.2	0.02	10.8 ±2.9	11.0 ± 3.4	0.4
K (mmol/L)	4.7±0.8	4.1± 0.7	0.07	4.7 ± 0.8	4.6 ± 0.8	0.08
Ca (mg/dL); corrected	9.2 ± 0.7	9.3 ± 0.7	0.4	9.2 ± 0.8	9.2 ± 0.7	0.5
P (mg/dL)	5.3 ± 1.4	5.3 ± 1.1	0.8	5.2 ± 1.4	5.3 ± 1.4	0.3
CaxP	49 ± 14	49 ± 11	0.8	48 ± 14	49 ± 14	0.4
iPTH (pg/mL)	257 (122, 485)	292 (128, 514)	0.8	235 (116, 464)	289 (140, 506)	0.06
hs-CRP (mg/L)	3.1 (1.2, 7.6)	2.4 (0.7, 6.2)	0.1	4.3 (1.6, 9.7)	2.3 (0.8, 5.4)	<0.001
Albumin (g/L)	4.1± 0.4	3.9 ± 0.3	0.01	4.0 ± 0.4	4.1 ± 0.4	0.02
Fetuin A (g/L)	0.45 (0.29, 0.7)	0.79 (0.65, 1.21)	<0.001	0.4 (0.27, 0.64)	0.62 (0.37, 0.82)	<0.001
GNRI	104.4 ± 9.7	102.8 ± 8.0	0.2	103.9 ± 9.7	104.7 ± 9.5	0.2
Medications (%)						
ESA	91	90	0.3	92	89	0.1
Active Vitamin D3	45	48	0.6	44	49	0.1
Phosphorus binder	85	90	0.4	90	84	0.5
Calcium-containing	64	67	0.5	60	54	0.3
Non-calcium-containing	21	23	0.3	30	30	0.4
Medications for osteoporosis	0.4	1.7	0.06	0.5	0.5	0.9
Anti-hypertensive agents	60	52	0.2	55	57	0.2

Abbreviations: CVD, cardiovascular disease; BP, blood pressure; Cre, creatinine; BMI, body mass index; CaxP, calcium phosphate product; iPTH, intact parathyroid hormone; hs-CRP, high-sensitive C-reactive protein; GNRI, geriatric nutritional risk index; ESA, erythropoiesis-stimulating agents.

*Note*: Conversion factors for units: hemoglobin in g/dL to g/L, ×10; serum calcium in mg/dL to mmol/L, ×0.2495; serum phosphate in mg/dL to mmol/L, ×0.3229; serum albumin in g/dL to g/L, ×10. No conversion is necessary for serum iPTH in pg/mL and ng/L; serum potassium in mEq/L and mmol/L.

### Outcomes

Overall, 177 of the participants experienced incident major fractures during a median of 3.4 years (IQR, 1.5–5.8 years) of follow-up. Thirty-seven participants had hip fractures, 10 had pelvic fractures, 20 had humeral fractures, 28 had distal forearm fractures, 32 had lower leg fractures and 50 had vertebral fractures. The incidence rate of major fractures was 3.29 per 100 person-years. The incidence rates in the lowest to highest fetuin A tertiles were 4.83, 3.21 and 1.45 per 100 person-years, respectively.

### Assessment of VC

A total of 365 participants had aortic arch calcification on the plain chest X-rays with different severity at entry, including 154, 124 and 87 participants in fetuin A tertile 1, 2 and 3, respectively. The number of participants with aortic arch calcification was significantly different in the fetuin A tertiles (P<0.001 by the chi-square test). One hundred and twenty-eight subjects with aortic arch calcification and 49 subjects without aortic arch calcification had major fractures. The incidence rates of major fractures in the participants with and without aortic arch calcification were 4.29 and 1.87 per 100 person-years, respectively.

### Associations between fetuin A level and fractures

In the multivariate Cox regression model, patients with higher fetuin A levels had a lower incidence of major fracture (adjusted HR, 0.28; 95% CI, 0.17–0.47, tertile 3 *vs*. tertile 1; adjusted HR, 0.54; 95% CI, 0.36–0.81, tertile 2 *vs*. tertile 1) in Model 1. Similarly, patients with higher fetuin A levels had a lower incidence of fractures in Model 2 and 3 (Table 3). Patients with aortic arch calcification at study entry had a higher risk of incident fractures (adjusted HR, 1.95; 95% CI, 1.34–2.84). Fetuin A levels and the presence of VC both independently predicted the risk of major fracture in our dialysis patients.

**Table 3:** Hazard ratios (HRs) of fetuin A tertiles in predicting the occurrence of major fractures using different Cox proportional hazard regression models.

Variables	Model 1	Model 2	Model 3
	Adjusted HR(95% CI) ^§^	Adjusted HR(95% CI)^§^	Adjusted HR(95% CI) ^§^
Fetuin A tertiles			
Fetuin A tertiles (3^rd^ *vs*. 1^st^ tertile)	0.28 (0.17‒0.47)	0.30 (0.18‒0.50)	0.34 (0.2‒0.57)
Fetuin A tertiles (2^nd^ *vs*. 1^st^ tertile)	0.54 (0.36‒0.81)	0.52 (0.34‒0.78)	0.53 (0.34‒0.81)
Other variables			
Gender (male *vs*. female)	0.68 (0.58‒0.92)	0.68 (0.49‒0.94)	0.72 (0.52‒0.99)
Age (every 1 year older)	1.03 (1.01‒1.04)	1.03 (1.01‒1.04)	1.02 (1.002‒1.03)
iPTH (every 100 unit increase)	-	1.05 (1.01‒1.1)	1.04 (1.008‒1.12)
hs-CRP (every 1 unit increase)	-	1.10 (1.02‒1.18)	1.11 (1.02‒1.19)
Aortic arch calcification (yes *vs*. no)	-	-	1.95 (1.34‒2.84)

Abbreviations: HR, hazard ratio; CI, confidence interval; iPTH, intact parathyroid hormone; hs-CRP, high-sensitive C-reactive protein, GNRI, geriatric nutritional risk index

^§^Adjusted for model 1: gender, age, dialysis vintage, previous fracture history, diabetes status/hypertension status, dialysis modality, smoking/alcohol status and patient cohort; model 2: factors in model 1 and hemoglobin level, intact parathyroid hormone, GNRI, CaxP and high-sensitive C-reactive protein (hs-CRP) levels; model 3: factors in model 2 and presence of aortic arch calcification.

### Recursive partitioning analysis

As we have shown in the analysis from the Cox proportional hazards model, fetuin A tertiles, gender, age, iPTH, hs-CRP and VC were among the significant predictive factors (Table 3) and they have selected to divide the patient population.

**Fig 2:**
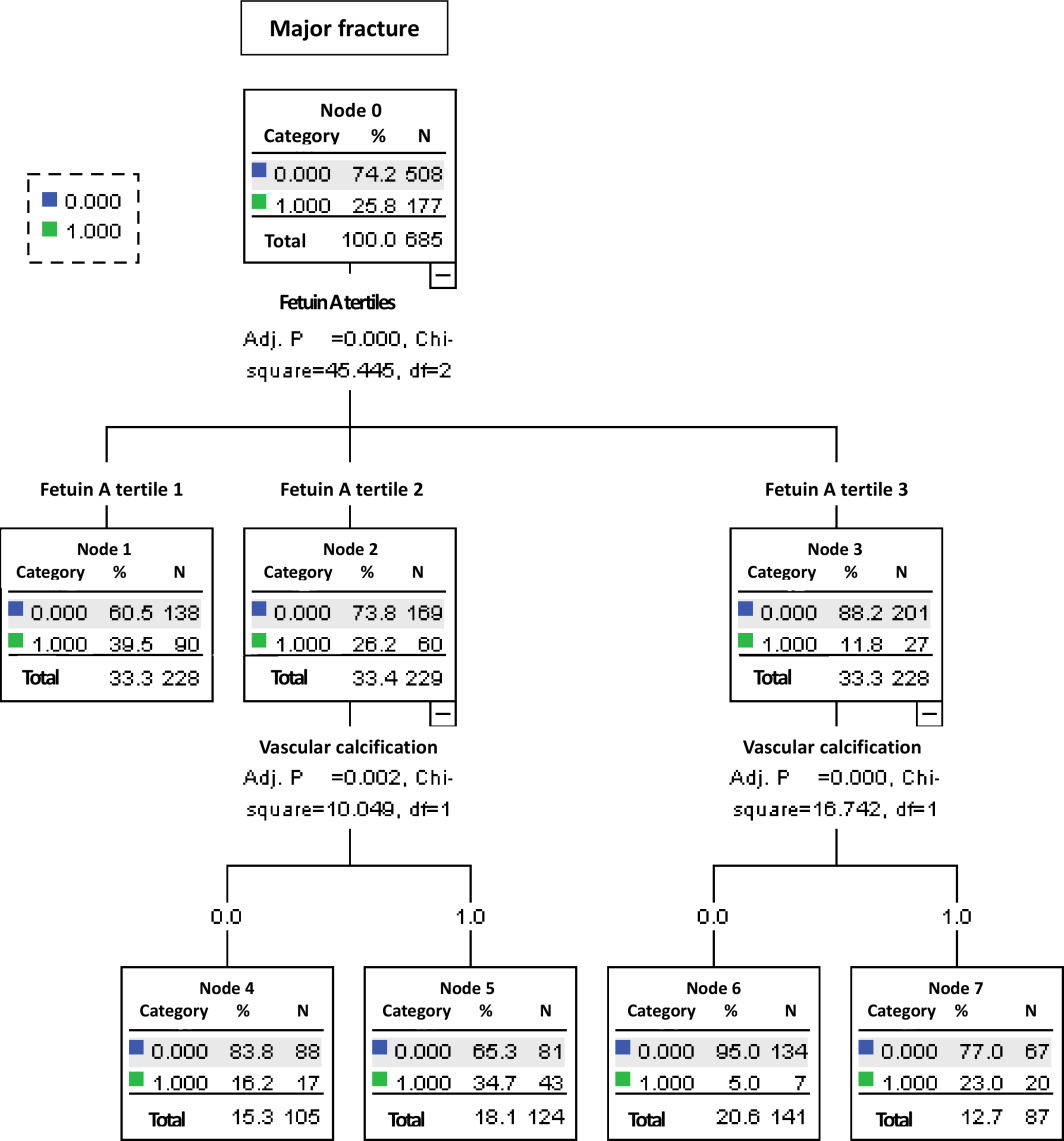
Classification tree based on recursive partitioning analysis.

**Fig 3:**
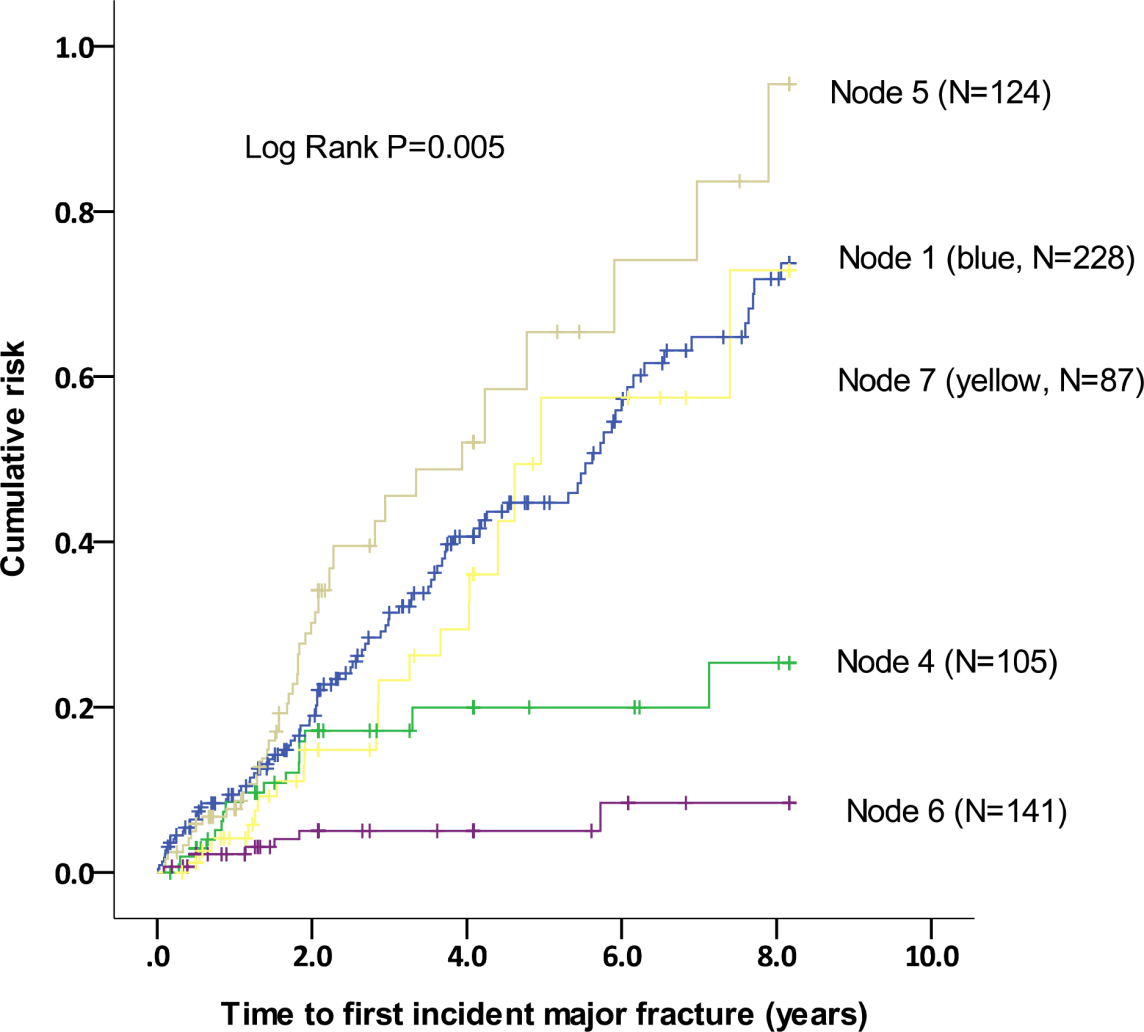
Kaplan-Meier survival curves for all patients, by risk group. Node 1: within fetuin A tertile 1, with or without VC; node 4: within fetuin A tertile 2, without VC; node 5: within fetuin A tertile 2, with VC; node 6: within fetuin A tertile 3, without VC and node 7: within fetuin A tertile 3, with VC.

Two hundred and twenty-eight patients were in the tertile 3 groups. For the patients in this group, presence of VC indicated a higher incidence of fracture, and among those within fetuin A tertile 2 (N = 229), presence of VC indicated a higher incidence of fracture as well. However, the subjects in the fetuin A tertile 1, presence of VC did not further differentiate the risk of major fracture (Fig 2). The 5 groups were ultimately defined (Node 1: within fetuin A tertile 1, with or without VC; node 4: within fetuin A tertile 2, without VC; node 5: within fetuin A tertile 2, with VC; node 6: within fetuin A tertile 3, without VC and node 7: within fetuin A tertile 3, with VC), and the Kaplan-Meier curves for these 5 groups are shown in Fig 3. Patients within the fetuin A tertile 3 and without VC had the lowest incidence of major fracture (Node 6).

### Associations between fetuin A level and VC

Because of the pathophysiological link between fetuin A and VC, as well as the very much interaction between fetuin A and VC (Fig 2), we further explored the interaction between fetuin A and VC in the prediction of incident fractures. We reported the interaction between fetuin A tertile and the presence of aortic arch calcification on the risk of incident major fracture in the Table 4. Either in patients with or without aortic arch calcification, those with higher fetuin A levels (tertile 2 and 3) had lower risk of incident fracture (P for interaction = 0.08, Table 4).

**Table 4:** Interaction between fetuin A tertile and the presence of aortic arch calcification on the risk of incident major fracture.

	Fetuin A tertile 1 (N = 228)		Fetuin A tertile 2 (N = 229)		Fetuin A tertile 3 (N = 228)			
	N with/without Major fracture	HR 95%CI	N with/without Major fracture	HR 95%CI	N with/without Major fracture	HR 95%CI	P for trend	P for interaction
With aortic arch calcification (n = 365)	65/89	1.0	43/81	0.54 (0.33‒0.87) P = 0.01	20/67	0.47 (0.26‒0.99) P = 0.05	P = 0.03	P = 0.08
Without aortic arch calcification (n = 320)	25/49	0.64 (0.39‒1.04) P = 0.07	17/88	0.29 (0.16‒0.55) P<0.001	7/134	0.11 (0.05‒0.26) P<0.001	P<0.001	

Adjusted for gender, age; dialysis vintage, diabetes/hypertension status, patient cohort, intact parathyroid hormone; body mass index, albumin and high-sensitive C-reactive protein (hs-CRP) levels, fetuin A tertile and presence of aortic arch calcification

Abbreviations: N, number; HR, hazard ratio; CI, confidence interval

### Comparison between PD and HD patients

Compared with PD patients, HD patients had lower fetuin A concentrations (0.47, 0.29‒ 0.7 *vs*. 0.79, 0.65‒1.21 g/L, P<0.001) (Table 2). The incidence rates of major fractures in our PD and HD patients were 0.43 and 3.41 per 100 person-years, respectively. HD patients had a significant higher risk to have incident fracture compared with PD patients (adjusted HR, 4.95; 95% CI, 1.19–20.68). In addition, the incidence rates of VC in our PD and HD patients were 3.49 and 6.78 per 100 person-years, respectively. Nonetheless, no significant difference was observed in the association of fetuin A level with the risk of major fractures between the two dialysis modalities (dialysis modality interaction analysis, P = 0.8).

### Sensitivity Analyses

When accounting for death as a potential informative event in competing risk models, the subhazard ratio (SHR) for major fracture continued to be significantly less for dialysis patients with higher fetuin A level examined in a fully adjusted model as well as those without the presence of aortic arch calcification (Table 5).

**Table 5:** Competing risk analysis of the relative hazard of major fracture by fetuin A tertile (death is the competing event).

Variables	Model 1	Model 2	Model 3
	Adjusted SHR(95% CI) ^§^	Adjusted SHR(95% CI)^§^	Adjusted SHR(95% CI) ^§^
Fetuin A tertiles			
Fetuin A tertiles (3^rd^ *vs*. 1^st^ tertile)	0.27 (0.16‒0.48)	0.27 (0.15‒0.49)	0.31 (0.17‒0.56)
Fetuin A tertiles (2^nd^ *vs*. 1^st^ tertile)	0.54 (0.35‒0.82)	0.49 (0.32‒0.78)	0.51 (0.32‒0.81)
Aortic arch calcification (yes *vs*. no)	-	-	1.88 (1.28‒2.77)

Abbreviations: SHR, subhazard ratio; CI, confidence interval; iPTH, intact parathyroid hormone; hs-CRP, high-sensitive C-reactive protein, GNRI, geriatric nutritional risk index

^§^Adjusted for model 1: gender, age, dialysis vintage, previous fracture history, diabetes status/hypertension status, dialysis modality, smoking/alcohol status and patient cohort; model 2: factors in model 1 and hemoglobin level, intact parathyroid hormone, GNRI, CaxP and high-sensitive C-reactive protein (hs-CRP) levels; model 3: factors in model 2 and presence of aortic arch calcification.

## Discussion

The main finding of this study is that the prevalent dialysis patients with a higher baseline fetuin A level had a lower long-term risk of incident major fractures regardless of gender, dialysis modality (HD or PD) and nutritional or inflammatory status; besides, patients with VC at study entry also had a higher risk to have incident fracture. Interestingly, the risk of incident fractures and aortic arch calcification declined in parallel with the increase in fetuin A level; however, lower fetuin A level and the presence of VC both independently predicted the higher risk of incident fracture in dialysis patients. In addition to the inhibitory property of VC and the strong predictive power of CV death, the fetuin A level also predicted the occurrence of major fractures in the dialysis patients.

Major fractures lead to high mortality and morbidity rates in dialysis patients,[3, 5] and therefore it is critical to identify high-risk patients who are susceptible to fragile fractures and to prevent potential fracture events in the long-term care of prevalent dialysis patients. Our results showed a high incidence of major fractures in the dialysis patients, around 3.29 per 100 person-years, which is nearly three times greater than that reported for elderly patients without CKD.[19] This incidence rate is similar to that presented in the Dialysis Outcomes and Practice Patterns Study [5] and the U.S. Renal Data System.[3] Of note, we found a link between fetuin A and incident fractures in our dialysis patients, that is, a higher fetuin A level predicted a lower incidence of fractures (Table 3, Fig 2). To the best of our knowledge, this is the first study to report an association between fetuin A level and incident fractures in dialysis patients. Therefore, fetuin A level in prevalent dialysis patients should be recognized as a potential marker to identify the dialysis patients who are at a higher risk of a major fracture.

In a landmark study using bone biopsies to estimate bone volume and multi-slice computed tomography to assess coronary artery calcification, low bone volume was found to be a significant risk factor for coronary calcification in HD patients.[10] In addition, low bone volume has been recognized as an important risk factor for the occurrence of fractures in both the general population [27–29] and dialysis patients.[30, 31] Therefore, it is reasonable to assume that dialysis patients with evident VC are more likely to experience a fracture. Our results have clearly shown that the dialysis patients with aortic arch calcification had a higher risk of fractures (Table 3, Figs 2 and 3). Specifically, in uremic milieu, dialysis patients tended to consume more fetuin A to prevent VC and therefore, dialysis subjects with baseline VC tended to have a lower fetuin A level concurrently. In an outstanding study which has shown that fetuin A is trafficked and exocytosed via exosome release in MV bodies. Interestingly, MV bodies containing exosomes were easily observed in vessels, especially in calcified MVs in dialysis patients. Factors that increase exosome release will promote vascular calcification, specifically under the condition of environmental calcium stress or fetuin A deficiency[16]. Therefore, the consequent generalized VC can be highly anticipated.

The features of osteoporosis in CKD patients are low trabecular bone volume and disrupted micro-architecture, but no abnormalities in mineralization or bone turnover[32]. Instead, bone loss is mostly from cortical bone in subjects with CKD mineral and bone disorder (CKD-MBD), and their iPTH, alkaline phosphatase, Klotho, sclerostin, and fetuin A levels are pronouncedly altered[33]. Due to the high prevalence of osteoporosis and CKD‒MBD in CKD subjects, both conditions are commonly existent simultaneously. But, in reality, the CKD–MBD is more complex than osteoporosis and CKD-MBD influences bone quality, contributes to high rates of fracture and most importantly, it results in VC in CKD patients. Although they both result in bone fragility, they do have different pathophysiology to destroy the bone. Osteoporosis is induced by excessive osteoclastic bone resorption in postmenopausal woman and subjects with aging process. But CKD-MBD is related to altered mineral metabolism and the imbalance of pro- and anti-calcification factors (such as fetuin A deficiency) which induced either high or low turnover bone disease in CKD subjects [33]. The *post hoc* analyses of data from crucial osteoporosis studies suggest that in patients with mild stage 3 CKD and normal iPTH, calcium and phosphate levels, conventional treatments for osteoporosis (such as bisphosphonates, teriparatide……) are effective to reduce fracture rates. Nevertheless, for patients with stage 4–5 CKD, the available evidence are insufficient to determine whether these medications are effective[33, 34]. Briefly speaking, low bone density and fractures induced by osteoporosis in patients with CKD differ from those with CKD-MBD.

In a recent investigation, Fink *et al* reported that serum fetuin A level had a positive association with areal BMD, but that there was no evidence of an association between fetuin A and the risk of clinical fractures in a large community cohort including 4714 elderly participants (>65 years of age)(the Cardiovascular Health Study).[19] A similar study including an even older cohort (70‒79 years of age), showed that a higher fetuin A level was correlated with higher BMD in older women.[18] Based on these reports, fetuin A level can predict BMD, but not the occurrence of major fractures in the elderly. These results seem to be in contrast to our findings; however, this may be due to several essential differences in the studies. First, our study was performed in patients undergoing dialysis rather than generally healthy elderly subjects. In the previous investigation, the dialysis patients had a lower serum fetuin A level than the age- and gender-matched cohort without CKD.[11] Our participants had a median level of fetuin A of 0.45 g/L (IQR 0.29‒0.73) which was lower than that in the Cardiovascular Health Study (0.47 g/L; IQR 0.41‒0.54). Furthermore, if we only selected participants older than 65 years in our study, we found that the median fetuin A level was even lower (0.36 g/L; IQR 0.24‒0.67). On the other hand, the fetuin A level has different clinical implications in patients undergoing dialysis and the general population. For example, dialysis patients with a lower fetuin A level have been reported to suffer from higher CV mortality,[11, 20, 35] and in the general population a lower fetuin A level has also been reported to lead to higher CV calcification.[36] However, in patients with diabetes, those with a higher fetuin A level have been reported to experience more CV complications.[37, 38] It is premature to recognize this phenomenon as another “reverse epidemiology”; nevertheless, the different impact of fetuin A level on incident fractures in different populations can rationally be anticipated. Second, our participants were much younger (59 ± 13 years) than those in the Cardiovascular Health Study (74.9 ± 5.3 years). According to our results, the risk of major fractures increased by 20~30% as the dialysis patients became older (every 10-year increase) (Table 3). In addition, a classical, large cohort study reported that patients in the general population older than 75 years had a higher odds of incident osteoporotic fractures.[39] Although it was underpowered to performed the subgroup analysis in this cohort because only 216 patients were older than 65 years old, we also found a trend of declining impacts of fetuin A levels on the incidence of major fracture in patients older than 65 year old (data not shown). And as we mentioned above, elderly CKD subjects have every likelihood that having osteoporotic fracture. Therefore, the impact of fetuin A level on fractures may be altered by the aging process.

Since fetuin A inhibits the VC process,[11, 40, 41] fetuin A and VC may be potential factors involved in the common pathway of the pathogenesis of fragile fractures in dialysis patients. However, little is known about the physiological link between fetuin A level and the occurrence of fractures. In vitro experiments, bone re-mineralization cannot successfully be performed in fetuin A-depleted serum, although it can be achieved when the serum is reconstituted with fetuin A.[12, 13] This suggests that fetuin A promotes bone mineralization in vitro. However, in fetuin A knock-out mice, the trabecular bone mass of cortical bone are unaffected by the absence of fetuin A; nevertheless, there is excess mineralization of the growth plate of long bone[17]. These conflict results from bench studies suggest that the pathophysiology between of fetuin A and bone mineralization remains unclear and it needs further works to be elucidated. In the view of epidemiological aspects, dialysis patients with evident VC had low bone volume[10] which led to the fragile fracture process, and concurrently, they have lower fetuin A levels as well due to a consumption process[42, 43], as also seen in our results (Table 2). We hypothesize that dialysis subjects with evident VC or having low fetuin A concentration would have more incident fracture owing to either their essential low bone volume or the lack of fetuin A to promote bone mineralization. In addition, sclerostin, a Wnt signaling pathway inhibitor, has been shown to be an important messenger in the cross-talk between bone and the vasculature, and the VC and fractures as well.[44, 45] In one phase II study, using romosozumab (a humanized monoclonal antibody to sclerostin) to treat 419 osteoporotic women, the results are promising; however the long-term (>12 months) effect is unknown.[46] Furthermore, a sclerostin inhibitor probably worsens VC in CKD rats [47] and may lead to deterioration of human renal function [33]. However, the association between fetuin A and sclerostin (or Wnt pathway) remains unknown. Therefore, further studies to investigate the potential biological pathway between fetuin A deficiency and the occurrence of incident fractures are warranted.

The strengths of this study are its prospective nature, balanced distribution of gender and available measurements of important confounding variables for outcome analysis, especially GNRI and hs-CRP levels and consistent results while performing both general Cox regression and competing risk models. However, there are some limitations to this study. First, the observational nature of this study precludes the conclusions of a causal relationship. Second, we did not check the BMD of the participants. Although the ability of BMD, as measured by dual-energy X-ray absorptiometry, in dialysis patients to predict the risk of fractures is weak,[31, 48] however, a recent study has confirmed the correlation between fetuin A level and BMD in the elderly. We could not fully confirm the correlation between fetuin A, BMD and fractures in dialysis patients from our results due to the lack of BMD measurements. Third, we only identified the presence of aortic arch calcification at entry and did not further quantify the severity of calcification. Dialysis patients with different severities of VC have been reported to have different fetuin A levels,[38, 42] and thereby potentially different risks of fractures. It would be more precise to quantify VC to better understand the interrelationship between fetuin A level, VC and fractures. Fourth, this was a single-center study, and all of the participants were treated by the same physicians and underwent uniform laboratory measurements during the observation period, which guaranteed the accuracy of our results. However, our conclusions cannot be generalized to other ethnicities. In summary, our results suggest that lower fetuin A level and the presence of aortic arch calcification can independently predict the long-term occurrence of incident fractures in prevalent dialysis patients.

## Supporting Information

S1 Fig Distribution of fetuin A concentrations in dialysis patients in the study (n = 685). A scatter plot of log hazard ratio (HR) of major incident fracture versus fetuin A concentration with Lowess smoothed function. The plot suggested nonlinear relationships; we therefore categorized patients into tertiles by fetuin A concentration for analyses.
(TIF)

## Abbreviations

BMD: bone mineral density
BMI: body mass index
CaxP: calcium phosphate product
CKD: chronic kidney disease
CV: cardiovascular
DM: diabetes mellitus
GNRI: geriatric nutritional risk index
HD: hemodialysis
HR: hazard ratio
hs-CRP: high sensitivity C-reactive protein
IQR: interquartile range
PD: peritoneal dialysis
iPTH: intact parathyroid hormone
VC: vascular calcification
