# Supplementary figures and images for: Relationship between Fetuin A, Vascular Calcification and Fracture Risk in Dialysis Patients
Source: PLoS One. 2016 Jul 11;11(7):e0158789. doi: 10.1371/journal.pone.0158789 (PMC4939952; doi:10.1371/journal.pone.0158789)

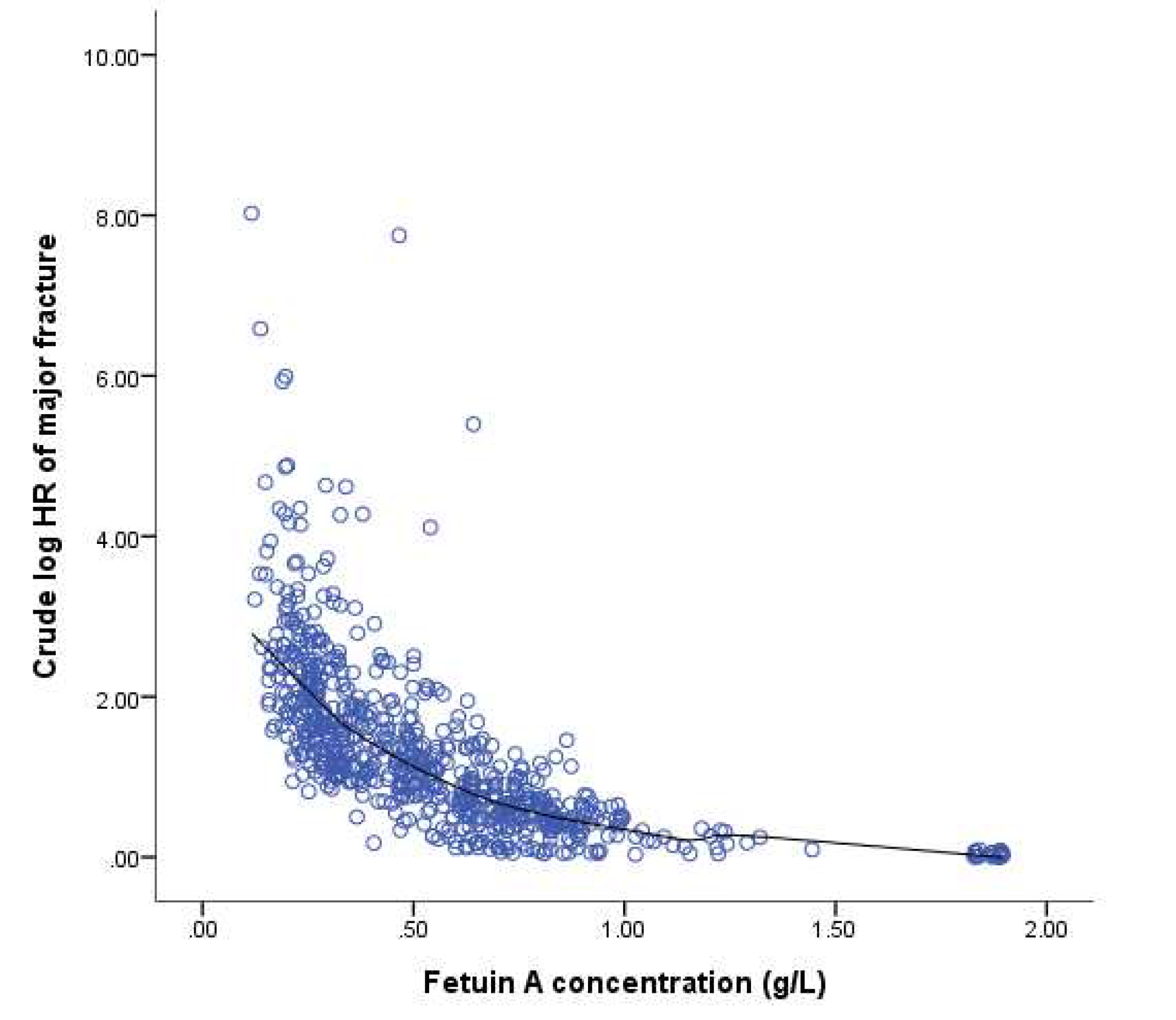

Supplement: S1 Fig — A scatter plot of log hazard ratio (HR) of major incident fracture versus fetuin A concentration with Lowess smoothed function. The plot suggested nonlinear relationships; we therefore categorized patients into tertiles by fetuin A concentration for analyses. (TIF) [file pone.0158789.s001.tif]
